# Intra-Tester and Inter-Tester Reliability of the Lachmeter When Measuring Knee Joint Laxity

**DOI:** 10.1155/2023/5583949

**Published:** 2023-08-08

**Authors:** Mikkel Oxfeldt, Anton B. Pedersen, Mette Hansen

**Affiliations:** Department of Public Health, Aarhus University, Aarhus, Denmark

## Abstract

Knee injuries are common among all age groups, and clinical knee examination is essential for the prognosis, follow-up, and rehabilitation process. The Lachmeter is a newly developed digitized modification of the Rolimeter, making it easier and faster for the test personnel to read the test result. In the present study, we aimed to evaluate the intra-tester and inter-tester reliability of the Lachmeter when testing healthy and traumatic knees. 24 healthy participants and a smaller sample of six ACL patients were examined with the Lachmeter by two intermediate testers and re-examined on a second visit within 21 days. All measurements were performed using two different grip techniques: a Lachman grip and an anterior drawer grip. Intra- and inter-tester reliability was evaluated using intra-class correlation coefficient (ICC), standard error of measurement (SEM), smallest detectable change (SDC), Student's paired *t*-test, and Bland–Altman plots. The results showed in healthy subjects poor to good intra-tester reliability (ICC range: −0.28–0.87, SEM range: 0.33–1.14 mm, and SDC range: 0.91–3.17 mm) and inter-tester reliability (ICC range: 0.41–0.87, SEM range: 0.27–0.67 mm, and SDC range: 0.75–1.87 mm). In ACL patients, intra-tester reliability was moderate to excellent (ICC range: 0.53–0.94, SEM range: 0.14–0.88 mm, and SDC range: 0.38–2.44 mm), with the exception of one measurement (ICC: 0.26 95% CI [−3.43; 0.89]), whereas inter-tester reliability was overall good (ICC range: 0.61–0.89, SEM range: 0.29–0.71 mm, and SDC range: 0.79–1.97 mm). Reliability measures between grip techniques indicated that the Lachman grip was more reliable than the anterior drawer grip. In conclusion, the Lachmeter showed variation between reliability measures, ranging from poor to good in healthy subjects and moderate to excellent in ACL patients. Future studies are needed to validate the Lachmeter against a gold-standard knee laxity assessment.

## 1. Introduction

Anterior cruciate ligament (ACL) injuries are common in sports requiring athletes to perform sudden stops and starts, jumping, and pivoting, such as handball and soccer [1]. In the USA alone, about 250000 ACL injuries are estimated to occur annually [2]. Treatment of ACL injuries varies depending on the patient's individual needs. However, whether the treatment is surgical or nonsurgical, rehabilitation is essential for an accelerated return to sport and prevention of knee re-injury [3]. A necessary tool for the clinical diagnosis and rehabilitation of ACL deficiency is the evaluation of ACL integrity. Arthrometry is a popular technique for assessing ACL integrity by quantifying knee displacement from an applied force [4]. Interestingly, research suggests combining arthrometry/laximetry with classic exam maneuverers of the knee surpasses the diagnostic abilities of MRI [5, 6]. Different types of arthrometers have been evaluated and compared in the literature regarding their reproducibility and validity. Overall, manual arthrometers are reported to be as good as automatic anthropometers [7]. Manual arthrometers are often cheaper, quicker, and lighter and include a more uncomplicated procedure for the test personnel than automated devices [7, 8]. The most popular manual arthrometer is the Rolimeter [6, 7, 9–12]. Reliability measures based on intra-class coefficients range from poor to excellent with an inter-tester reliability interval of 0.55–0.95 [10, 12, 13] and an intra-tester interval of 0.24–1.0 [8, 12, 13]. However, one problematic feature of the Rolimeter is the analogue gauge of the device, which is difficult for the tester to interpret accurately. Accordingly, reliability and validity often depend upon examiner familiarity and skills [6].

The Lachmeter (Lachmeter®, Lachmeter Company, Ribeirao Preto, Sao Paulo, Brazil) is a newly developed digitized modification of the Rolimeter in which the analogue gauge is replaced with a digital display making it easy to read for the test personnel. Today, only one study by Krautter et al. has compared a digitized Rolimeter to the traditional Rolimeter by testing 50 participants comprised of acute ACL patients, ACL-treated patients, and controls [11]. They demonstrated that the digitized Rolimeter offers easier, faster, and more precise measurements than the manual Rolimeter, especially in postoperative examinations of ACL reconstructions [11]. Surprisingly, even though the mechanics of the two devices were the same, Krautter et al. used different grip techniques for the two devices, i.e., anterior drawer grip with the digitized Rolimeter and Lachman grip with the classic Rolimeter. Thus, the difference reported between the two devices may reflect differences between the anterior drawer grip and Lachman grip, rather than differences between the devices. In addition, no study has investigated the intra-tester and inter-tester reliability of the Lachmeter. Therefore, the present study aimed to evaluate the intra-tester and inter-tester reliability of the Lachmeter when testing healthy and ACL-injured knees. We hypothesized that the Lachmeter would be a reliable tool for evaluating knee displacement.

## 2. Methods

### 2.1. Design

The present study was conducted as a prospective test-retest study where two testers measured anterior tibial translation using the Lachmeter in healthy subjects and conservatively treated ACL patients. Anterior tibial translation was measured with two different grip techniques: a Lachman grip and an anterior drawer grip. Measurements were collected by both testers on the first day of testing and repeated on a second day within 21 days. For the ACL-injured group, the testers were blinded in relation to which leg was injured. Both testers were considered intermediate users of the Lachmeter since they had received training from a clinician and had practiced regularly for two months (approximately 15 subjects, measured 1–3 times) leading up to the data collection.

### 2.2. Participants

Twenty-four healthy young participants were recruited to participate from the local university, and six ACL patients were recruited to participate from a local rehabilitation center (Table 1). The ACL-injured group consisted of patients diagnosed with an ACL injury within the last 12 months. All patients had received a full diagnosis from a physician and confirmed MR imaging at Aarhus University Hospital. Exclusion criteria for the patients were as follows: (1) ACL reconstruction and (2) pronounced pain, making it impossible to measure knee laxity. Exclusion criteria for the healthy control group included a current or previous ACL injury or other injuries, which hindered the measure of knee laxity without pain. All participants were given verbal and written information about the study before they gave their written consent.

### 2.3. Procedure

**Figure 1:**
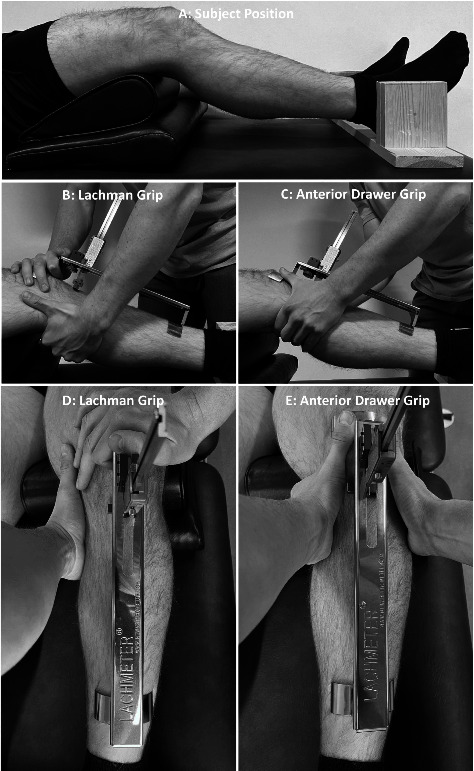
Subject starting position (a), Lachman grip test procedure (b, d), and anterior drawer grip test procedure (c, e).

Arriving on the first test day, the participants completed a short questionnaire about their body characteristics, knee injury history, and weekly physical training. Hereafter, the participants were placed in a supine position with their feet planted in a customized foot stand to avoid any changes to the rotation of the hip joint during testing. The Lachmeter comes with a small firm pillow, which was placed under the femur resulting in a 5–10° knee flexion. However, since the most accurate measurements of the ACL stiffness are made at approximately 20° [6], an additional pillow was placed under the femur to achieve 20° knee flexion (see Figure 1). Before placing the Lachmeter on the leg, the examiner marked the tuberosity of the tibia. The Lachmeter was then placed medially on the patella and distally on the anterior part of the tibia with the moveable reader-bar on the tuberosity tibia mark. Hereafter, the sliding reader-bar was pressed smoothly down until the feeling pad touched the tuberosity of the tibia and the digital display was reset. Then, the examiner slowly applied a maximum manual force facilitating the anterior displacement of the tibia using one of two different handgrip techniques (the Lachman grip and the anterior drawer grip). The Lachman grip was always performed before the anterior drawer grip.

Before each measurement, the participants were asked to relax their lower body. Four measurements were performed on both knees using the two different techniques, resulting in sixteen measurements per subject per day. The first measurement was used as an examiner “warm-up” and as subject familiarization to avoid the fear of pain during the test. Thus, the first measurement was excluded from the analysis, and a mean value of the three last measurements on each knee using each technique was used for further analysis.

All measurements were repeated on test day 2, separated by 2–21 days from test day 1. The individual participants were tested approximately at the same time of day ± 2 hours.

To avoid any bias within the intra- and inter-tester reliability measures, Tester 1 reset the testing conditions before Tester 2 arrived, and they were never in the room while the other tester tested a subject. Furthermore, measurements were written down on paper and hidden away, meaning both testers were blinded to each other's and previous test results.

### 2.4. Lachman Grip

The examiner placed one hand dorsally on the calf in line with the mark of tibia tuberosity (right hand for right leg and conversely). This hand slowly pulled the tibia in a ventral direction, while the other hand stabilized the proximal plate on the medial patella by applying mild pressure with the thumb located on the frame slightly below the reader-bar as illustrated in Figure 1. This grip technique is similar to the Lachman test [14] and will be referred to as the Lachman grip in the present article.

### 2.5. Anterior Drawer Grip

Executing the second technique, the examiner placed both thumbs on top of the proximal plate on the medial patella to apply stability and counterforce during the measures. The fingers were placed dorsally on the calf under the mark of tuberosity tibia. During the measurements, both hands were used to apply an anterior low-velocity force (see Figure 1). This grip technique is similar to anterior drawer test [14] and will be referred to as the anterior drawer grip in the present article.

### 2.6. Statistics

Data were tested for normal distribution by a normality and log normality test (D'Agostino and Pearson test) before any statistical analyses were performed. Unpaired Student's *t*-tests were carried out to analyze differences between groups at baseline. To determine the intra- and inter-reliability of the Lachmeter, intra-class correlation coefficients (ICC) were calculated. A one-way random effects model (subject as random effects) of absolute agreement was used to determine intra-tester reliability (differences from day 1 to day 2 for the individual tester). A two-way random effects model (subject and tester as random effects) of absolute agreement was used to determine inter-rater reliability (differences between the two testers). These analyses were performed for both legs and the side-to-side mean values. The interpretation of the ICC outcomes followed the clinical standards [15]: >0.90 = excellent; 0.75 to 0.90 = good; 0.50 to 0.75 = moderate; and <0.50 = poor reliability. Student's paired *t*-tests were performed to test for the difference between test 1 and test 2, as well as difference between Tester 1 and Tester 2. Standard error of measurement (SEM) was calculated from the within-subject standard deviation and representative ICC using the following equation: SEM = SD × square root (1 − ICC). Smallest detectable change (SDC) was calculated from the SEM using the following equation: SDC = SEM × 1.96 × square root [7]. Side-to-side difference was calculated as the difference between the dominant and non-dominant leg measured in millimeter. All data are presented as mean ± standard deviation (SD). The statistically significant level was set at *p* ≤ 0.05. Data were analyzed using Stata version 17 software (StataCorp, College Station, TX, USA) and Bland–Altman plots using GraphPad Prism version 8.0.2 (GraphPad Software, Inc.).

## 3. Results

Twenty-four healthy subjects (33.3% females) and six ACL patients (50% females) were eligible to be included in the analysis. A group difference in age was observed between subjects and ACL patients, but no other significant group differences were observed regarding height, weight, or physical training (Table 1). One of the healthy participants only participated in the first test. Accordingly, these data are only included in the inter-tester reliability analysis.

### 3.1. Intra-Tester Reliability for Healthy Subjects and ACL Patients

For healthy subjects, the test-retest reliability for Tester 1 ranged from moderate to good and that for Tester 2 was moderate using both types of grip (Table 2). When evaluating side-to-side differences between the dominant and non-dominant leg from day 1 vs. 2, the Lachman grip demonstrated poor reliability for both testers, whereas the anterior drawer grip demonstrated moderate reliability for Tester 1 and poor reliability for Tester 2. SEM and SDC were generally smallest for the Lachman grip (SEM range: 0.33–0.85 mm and SDC range: 0.91–2.73 mm) compared to the anterior drawer grip (SEM range: 0.53–1.14 mm and SDC range: 1.46–3.17 mm) for both testers (Table 2).

For ACL patients, the test-retest reliability for Tester 1 and Tester 2 ranged from good to excellent for both grip types (Table 3). When evaluating side-to-side differences between the dominant and non-dominant leg from test day 1 vs. 2, the Lachman grip demonstrated moderate to excellent reliability for both testers, whereas the anterior drawer grip demonstrated poor to moderate. SEM and SDC were generally smallest for the Lachman grip (SEM range: 0.15–0.68 mm and SDC range: 0.42–1.73 mm) compared to the anterior drawer grip (SEM range: 0.14–0.95 mm and SDC range: 0.38–2.62 mm) for both testers (Table 3).

### 3.2. Inter-Tester Reliability for Healthy Subjects and ACL Patients

For the healthy subjects, the overall reliability between the two testers (between-tester variation) was good for measurements of the dominant and non-dominant leg using the Lachman grip but poor when evaluating side-to-side differences (Table 4). For the anterior drawer grip, reliability was moderate to good, but side-to-side differences showed poor reliability (Table 4). SEM and SDC favored the Lachman grip (SEM range: 0.27–0.53 mm and SDC range: 0.75–1.47 mm) over the anterior drawer grip (SEM range: 0.61–0.67 mm and SDC range: 1.69–1.87 mm).

For the ACL patients, the reliability between the two testers was overall good. Nevertheless, side-to-side differences using the anterior drawer grip showed moderate reliability (Table 5). SEM and SDC tended to favor the Lachman grip (SEM range: 0.29–0.66 mm and SDC range: 0.79–1.83 mm) over the anterior drawer grip (SEM range: 0.29–0.71 mm and SDC range: 0.81–1.97 mm).

### 3.3. Bland–Altman Plots and Limits of Agreement

**Figure 2:**
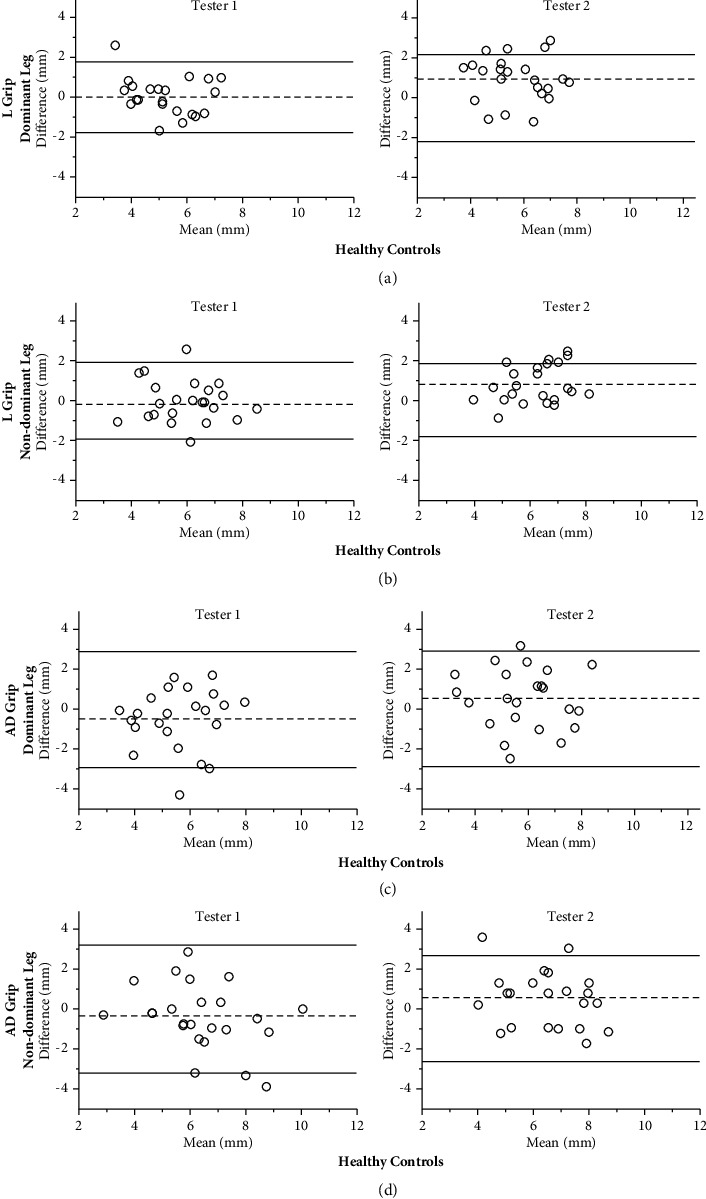
Bland–Altman plots of the difference between day 1 and day 2 in healthy subjects with the Lachman grip and anterior drawer grip of the dominant and non-dominant leg for Tester 1 and Tester 2, respectively. The central dotted line represents the mean differences between day 1 and day 2, whereas the upper and lower lines represent the upper and lower 95% limits of agreement (mean differences ± 1.96 SD of the differences). L grip, Lachman grip; AD grip, anterior drawer grip. (a) L grip dominant leg. (b) L grip non-dominant leg. (c) AD grip dominant leg. (d) AD grip non-dominant leg.

Figure 2 shows Bland–Altman plots and limits of agreement (LOA) in healthy subjects, illustrating the agreement between test day 1 and 2, as well as Tester 1 and Tester 2, for each knee and each technique utilized. Consistent with the other reliability estimates, the measurement bias (mean difference) was smaller, and LOA were narrower for the Lachman grip compared to the anterior drawer grip.

**Figure 3:**
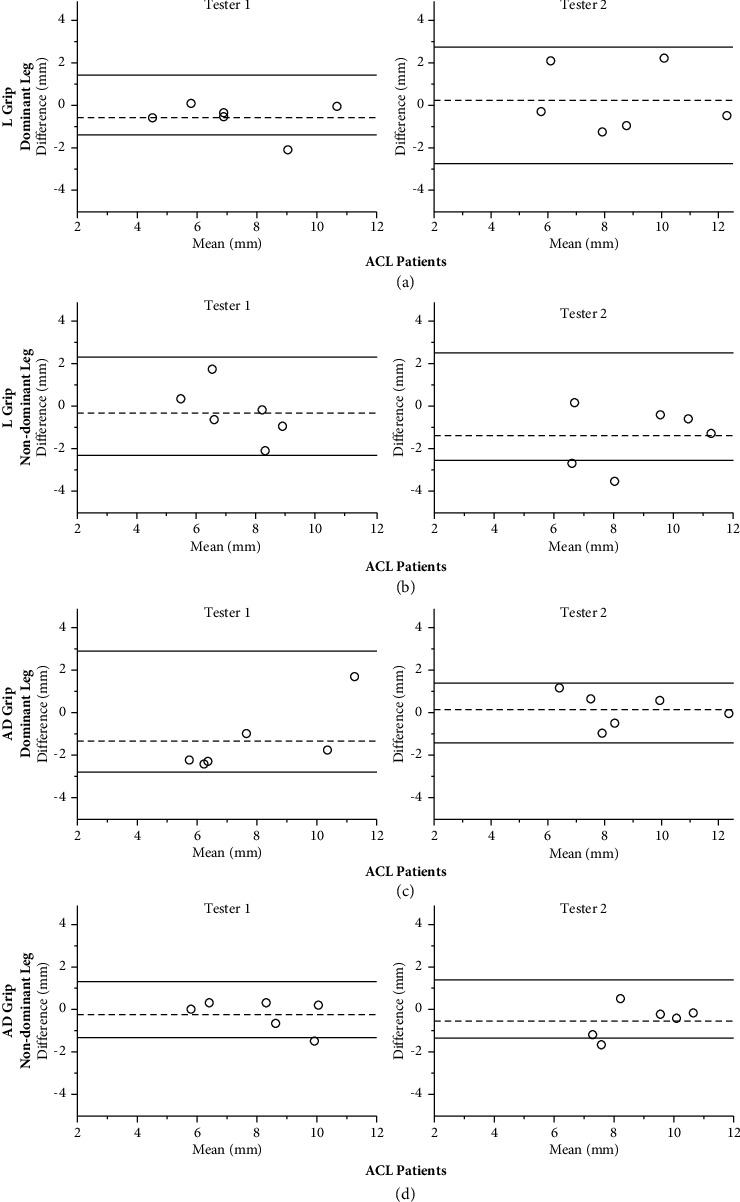
Bland–Altman plots of the difference between day 1 and day 2 in ACL patients with the Lachman grip and anterior drawer grip of the dominant and non-dominant leg for Tester 1 and Tester 2, respectively. The central dotted line represents the mean differences between day 1 and day 2, whereas the upper and lower lines represent the upper and lower 95% limits of agreement (mean differences ± 1.96 SD of the differences). L grip, Lachman grip; AD grip, anterior drawer grip. (a) L grip dominant leg. (b) L grip non-dominant leg. (c) AD grip dominant leg. (d) AD grip non-dominant leg.

Figure 3 shows Bland–Altman plots and limits of agreement (LOA) in ACL patients. In contrast to healthy subjects, no systematic difference between grips was observed.

### 3.4. Healthy Subjects vs. ACL Patients

Tester 1 obtained a significantly higher mean side-to-side difference for the ACL group compared to the control group using the Lachman grip (*p* < 0.01) (Table 6). The same tendency was observed using the anterior drawer grip (*p*=0.06). Likewise, Tester 2 measured a significantly higher mean side-to-side difference for the ACL group compared to the control group when using both techniques. No difference was observed between testers (*p* > 0.05).

## 4. Discussion

The present study evaluated intra-tester and inter-tester reliability of the Lachmeter using two different grip techniques. The main findings of the present study were that the Lachmeter in healthy subjects showed poor to good intra-tester and inter-tester reliability. However, based on the six included ACL patients, intra-tester reliability was generally moderate to excellent and inter-tester reliability was overall good. Furthermore, reliability estimates were generally better using the Lachman grip than the anterior drawer grip. To our knowledge, this study is the first to evaluate the reliability of the Lachmeter.

### 4.1. Intra-Tester Reliability

Intra-tester reliability has not previously been reported in the literature for the Lachmeter. Thus, our data are considered the first to demonstrate poor to good intra-tester reliability in healthy subjects and moderate to excellent reliability in ACL patients between day-to-day measures with ICC ranging from −0.28–0.87 in healthy subjects and 0.26–0.97 in ACL patients. In comparison, studies investigating intra-tester reliability of the Rolimeter have found an overall good test-retest reliability [8, 10, 13]. Previous reliability studies of the Rolimeter have pointed out that the skill and consistency of the tester are important since it is difficult to standardize the force or technique applied during a Lachman test [6, 10, 13]. In Muellner et al., two experienced orthopedic surgeons and one intermediate medical student evaluated ten healthy participants twice with the Rolimeter [13]. The participants were tested at 20–30° knee flexion using the Lachman grip. The intra-tester reliability analysis of measurements on the same knee showed best values for the experienced testers (*r* = 0.65 and *r* = 0.72), corresponding to a moderately good correlation between the means of each tester's first and second reading, whereas the correlation for the intermediate user was moderate (*r* = 0.55). In summary, previous literature evaluating the intra-tester reliability of the Rolimeter demonstrates moderate-to-excellentintra-tester reliability, which is in agreement with our findings of the Lachmeter, at least in ACL patients. However, the results for the Rolimeter seem to depend on the tester's experience. Accordingly, the Rolimeter is often critiqued for the analogue gauge of the device, which is difficult for the tester to interpret accurately, especially with low experience. The Lachmeter uses a digital display, making it faster and easier to read for the test personnel, which should theoretically reduce day-to-day variability and the experience required by the tester. In line with the latter, even though both testers in the present study were considered intermediate users (i.e., had several weeks of experience with the Lachmeter), intra-tester reliability was found to be good to excellent for some measures when using the Lachman grip, which is greater than reliability scores performed by intermediate users of the Rolimeter [10, 13]. However, large variance was also observed between the different measurements, with some showing poor reliability and others showing excellent reliability. This highlights the difficulties and constraints of manually assessing knee laxity, as it is highly sensitive to the force applied by the tester during measurements. Therefore, it is crucial to emphasize the significance of tester experience when performing manual assessments of knee laxity.

### 4.2. Inter-Tester Reliability

When comparing the difference between the two testers, we observed moderate to good reliability for the measurements of the dominant and non-dominant tibial translation and poor to good reliability for the side-to-side measurements between the two testers. Studies investigating inter-tester reliability of the Rolimeter have found a larger variation in the inter-tester reliability than in the present study [10, 12, 13]. Moreover, inter-tester reliability of the Rolimeter has ranged from low to excellent in previous studies with different tester experience [10, 12, 13]. In comparison, our results of the Lachmeter are within the better range of previously described inter-tester reliability for the Rolimeter, suggesting that results between testers are comparable when using the Lachmeter.

### 4.3. Influence of Grip Technique

In the present study, we investigated if the Lachman grip in comparison to the anterior drawer grip would influence the measurements. Although small differences were observed between grip techniques, the best reliability measures were found using the Lachman grip. In line with our findings, a number of systematic reviews [14, 16–18] have investigated the validity between the Lachman test and the anterior drawer test performed manually and overall concluded that the Lachman test is superior to the anterior drawer test, due to lower sensitivity and specificity for the latter grip test procedure. Specifically, a meta-analysis by Benjaminse et al. analyzed 28 studies that assessed the accuracy of the anterior drawer test and the Lachman test for diagnosing ACL ruptures. Their results showed that the anterior drawer test had a sensitivity of 49% and a specificity of 58%, which is considered unacceptably low, especially during the acute phase of an ACL injury [14]. Accordingly, we experienced some challenges when using the anterior drawer grip. We observed that the measurement could be compromised if the subject's legs were too thick, or if the tester's hands were relatively too small, making it hard to reach and stabilize the Lachmeter while applying the force. The convex pad on the patella could thereby slightly slip, resulting in a false measurement. Collectively, based on our results and previous literature [14, 16–18], we recommend using the Lachman grip when evaluating knee laxity.

### 4.4. Strength and Limitations

A strength of the present approach to testing the Lachmeter was that many methodological considerations (i.e., subject position, device placement, technical standardization, ACL inclusion criteria, and foot stand to minimize hip rotation) had been taken into account. However, even though the testers were blinded for each other's and their own results, blinding for group assignments was not possible due to the testing location. This may have introduced a risk of bias since the testers knew if they tested an ACL patient or not.

A major limitation of the present study is the low number of ACL patients included. This limits the interpretation of our data in terms of describing the validation and sensitivity of the Lachmeter in the clinical diagnosis of ACL injuries. We recommend that future studies include a larger number of ACL patients to verify the sensitivity of the Lachmeter for clinical use.

## 5. Conclusion

Lachmeter measurements carried out by two intermediate users of the device demonstrated poor to good intra-tester and inter-tester reliability in healthy subjects. In a smaller sample of ACL patients, intra-tester reliability was moderate to excellent and inter-tester reliability was overall good. Reliability estimates were generally better when the device was used with the Lachman grip compared to the anterior drawer grip. Future studies are needed to validate the Lachmeter against a gold-standard knee laxity assessment.

## Figures and Tables

**Table 1:** Subject characteristics.

	Control, *n* = 24	ACL patients, *n* = 6	*p* value
Age (y)	24.3 ± 1.2	44.7 ± 18.3	<0.001
Height (cm)	180.0 ± 8.6	172.2 ± 9.9	0.07
Weight (kg)	77.6 ± 12.0	74.5 ± 10.0	0.58
Physical training (hrs/week)	7.6 ± 4.0	4.3 ± 3.3	0.07

ACL: anterior cruciate ligament. Values are presented as mean ± standard deviation (SD). *p* values correspond to tests for difference between groups (unpaired *t*-test).

**Table 2:** Intra-tester reliability for measurements of anterior tibial translation (mm) in healthy subjects (*n* = 23) performed on 2 separate days.

Tester	Grip	Leg	Dif. between day 1 and day 2 mean (SD) [95% CI]	Between test *p*value	Average ICC [95% CI]	Interpretation of ICC	SEM	SDC
Tester 1	L grip	Non-dominant day 1 vs. 2	0.17 (1.00) [−0.27; 0.60]	0.44	0.83 [0.61; 0.93]	Good reliability	0.41	1.14
		Dominant day 1 vs. 2	−0.01 (0.91) [−0.40; 0.38]	0.96	0.87 [0.69; 0.94]	Good reliability	0.33	0.91
	AD grip	Non-dominant day 1 vs. 2	0.41 (1.68) [−0.32; 1.14]	0.26	0.71 [0.33; 0.88]	Moderate reliability	0.90	2.50
		Dominant day 1 vs. 2	0.48 (1.52) [−0.18; 1.14]	0.14	0.59 [0.05; 0.83]	Moderate reliability	0.97	2.70
	L grip	Side-to-side day 1 vs. 2	0.42 (1.08) [−0.05; 0.88]	0.08	0.17 [−0.91; 0.65]	Poor reliability	0.98	2.73
	AD grip	Side-to-side day 1 vs. 2	0.11 (0.93) [−0.29; 0.51]	0.58	0.68 [0.26; 0.86]	Moderate reliability	0.53	1.46

Tester 2	L grip	Non-dominant day 1 vs. 2	−0.79 (0.95) [−1.20; −0.37]	<0.001	0.68 [0.25; 0.86]	Moderate reliability	0.54	1.49
		Dominant day 1 vs. 2	−0.97 (1.10) [−1.45; −0.49]	<0.001	0.62 [0.11; 0.84]	Moderate reliability	0.68	1.88
	AD grip	Non-dominant day 1 vs. 2	−0.58 (1.39) [−1.18; 0.02]	0.06	0.72 [0.34; 0.88]	Moderate reliability	0.74	2.04
		Dominant day 1 vs. 2	−0.50 (1.50) [−1.15; 0.15]	0.13	0.70 [0.31; 0.87]	Moderate reliability	0.82	2.28
	L grip	Side-to-side day 1 vs. 2	0.26 (0.91) [−0.13; 0.65]	0.18	0.13 [−1.02; 0.63]	Poor reliability	0.85	2.35
	AD grip	Side-to-side day 1 vs. 2	−0.42 (1.01) [−0.86; 0.02]	0.06	−0.28 [−1.98; 0.45]	Poor reliability	1.14	3.17

L grip: Lachman grip; AD grip: anterior drawer grip; ICC: intra-class correlation coefficient; SEM: standard error of measurement. Anterior tibial translation was measured in mm, and the difference between day 1 and 2 is presented in healthy subjects (*n* = 23). *p* values correspond to the results from using Student's paired *t*-test to test for differences between test 1 and test 2.

**Table 3:** Intra-tester reliability for measurements of anterior tibial translation (mm) in ACL patients (*n* = 6) performed on 2 separate days.

Tester	Grip	Leg	Dif. between day 1 and day 2 mean (SD) [95% CI]	Between test *p*value	Average ICC [95% CI]	Interpretation of ICC	SEM	SDC
Tester 1	L grip	Non-dominant day 1 vs. 2	0.30 (1.28) [−1.04; 1.63]	0.59	0.80 [−0.21; 0.97]	Good reliability	0.57	1.59
		Dominant day 1 vs. 2	0.59 (0.78) [−0.24; 1.41]	0.13	0.96 [0.74; 0.99]	Excellent reliability	0.16	0.43
	AD grip	Non-dominant day 1 vs. 2	0.20 (0.72) [−0.56; 0.95]	0.53	0.96 [0.77; 0.99]	Excellent reliability	0.14	0.40
		Dominant day 1 vs. 2	1.37 (1.58) [−0.29; 3.02]	0.09	0.82 [−0.09; 0.98]	Good reliability	0.67	1.86
	L grip	Side-to-side day 1 vs. 2	0.49 (0.67) [−0.21; 1.20]	0.13	0.95 [0.69; 0.99]	Excellent reliability	0.15	0.42
	AD grip	Side-to-side day 1 vs. 2	−0.47 (1.61) [−2.17; 1.23]	0.51	0.70 [−0.82; 0.96]	Moderate reliability	0.88	2.44

Tester 2	L grip	Non-dominant day 1 vs. 2	1.35 (1.42) [−0.14; 2.84]	0.07	0.77 [−0.35; 0.97]	Good reliability	0.68	1.89
		Dominant day 1 vs. 2	−0.25 (1.54) [−1.86; 1.37]	0.71	0.92 [0.51; 0.99]	Excellent reliability	0.44	1.21
	AD grip	Non-dominant day 1 vs. 2	0.58 (0.77) [−0.23; 1.39]	0.13	0.90 [0.38; 0.99]	Good reliability	0.24	0.67
		Dominant day 1 vs. 2	−0.18 (0.80) [−1.02; 0.65]	0.60	0.97 [0.81; 0.99]	Excellent reliability	0.14	0.38
	L grip	Side-to-side day 1 vs. 2	−0.44 (1.12) [−1.61; 0.74]	0.38	0.69 [−0.85; 0.95]	Moderate reliability	0.62	1.73
	AD grip	Side-to-side day 1 vs. 2	0.14 (1.10) [−1.02; 1.29]	0.78	0.26 [−3.43; 0.89]	Poor reliability	0.95	2.62

L grip: Lachman grip; AD grip: anterior drawer grip; ICC: intra-class correlation coefficient; SEM: standard error of measurement. Anterior tibial translation was measured in mm, and the difference between day 1 and 2 is presented in ACL patients (*n* = 6). *p* values correspond to the results from using Student's paired *t*-test to test for differences between test 1 and test 2.

**Table 4:** Inter-tester reliability for measurements of anterior tibial translation (mm) in healthy subjects (*n* = 23) performed by two testers.

Grip	Leg	Dif. between testers mean (SD) [95% CI]	*p* value	Average ICC [95% CI]	Interpretation of ICC	SEM	SDC
L grip	Non-dominant Tester 1 vs. 2	−0.30 (0.83) [−0.65; 0.06]	0.10	0.84 [0.63; 0.93]	Good reliability	0.33	0.92
	Dominant Tester 1 vs. 2	−0.40 (0.75) [−0.72; 0.08]	0.02	0.87 [0.65; 0.95]	Good reliability	0.27	0.75

AD grip	Non-dominant Tester 1 vs. 2	0.04 (1.29) [−0.52; 0.60]	0.89	0.77 [0.46; 0.90]	Good reliability	0.62	1.71
	Dominant Tester 1 vs. 2	−0.29 (1.25) [−0.83; 0.25]	0.28	0.71 [0.33; 0.88]	Moderate reliability	0.67	1.87

L grip	Side-to-side Tester 1 vs. 2	0.03 (0.69) [−0.27; 0.33]	0.82	0.41 [−0.44; 0.75]	Poor reliability	0.53	1.47

AD grip	Side-to-side Tester 1 vs. 2	0.28 (0.80) [−0.06; 0.63]	0.10	0.42 [−0.27; 0.75]	Poor reliability	0.61	1.69

L grip: Lachman grip; AD grip: anterior drawer grip; ICC: intra-class correlation coefficient. Anterior tibial translation was measured in mm, and the difference between Tester 1 and 2 is presented in healthy subjects (*n* = 24). *p* values correspond to the results from using Student's paired *t*-tests to test for differences between Tester 1 and Tester 2.

**Table 5:** Inter-tester reliability for measurements of anterior tibial translation (mm) in ACL patients (*n* = 6) performed by two testers.

Grip	Leg	Dif. between testers mean (SD) [95% CI]	*p* value	Average ICC [95% CI]	Interpretation of ICC	SEM	SDC
L grip	Non-dominant Tester 1 vs. 2	−1.43 (1.04) [−2.52; −0.33]	0.02	0.76 [−0.28; 0.97]	Good reliability	0.51	1.41
	Dominant Tester 1 vs. 2	−1.20 (1.60) [−2.90; 0.47]	0.13	0.83 [0.02; 0.97]	Good reliability	0.66	1.83

AD grip	Non-dominant Tester 1 vs. 2	−0.81 (0.84) [−1.69; 0.07]	0.06	0.88 [0.08; 0.98]	Good reliability	0.29	0.81
	Dominant Tester 1 vs. 2	−0.80 (1.27) [−2.14; 0.54]	0.18	0.89 [0.36; 0.98]	Good reliability	0.42	1.17

L grip	Side-to-side Tester 1 vs. 2	−0.44 (0.86) [−1.35; 0.46]	0.26	0.89 [0.36; 0.98]	Good reliability	0.29	0.79

AD grip	Side-to-side Tester 1 vs. 2	0.48 (1.14) [−0.72; 1.68]	0.35	0.61 [−1.36; 0.94]	Moderate reliability	0.71	1.97

L grip: Lachman grip; AD grip: anterior drawer grip; ICC: intra-class correlation coefficient. Anterior tibial translation was measured in mm, and the difference between Tester 1 and 2 is presented in ACL patients (*n* = 6). *p* values correspond to the results from using Student's unpaired *t*-tests to test for differences between Tester 1 and Tester 2.

**Table 6:** Comparison of mean side-to-side difference between healthy subjects (*n* = 23) and ACL patients (*n* = 6).

Tester	Grip	Control side-to-side mean (SD) [95% CI]	ACL side-to-side mean (SD) [95% CI]	*p* value
Tester 1	L grip	1.07 (0.84) [0.82; 1.32]	2.23 (1.72) [1.14; 3.32]	<0.01
	AD grip	1.41 (0.93) [1.14; 1.69]	2.08 (1.57) [1.09; 3.08]	0.06

Tester 2	L grip	1.04 (0.68) [0.84; 1.24]	2.67 (1.16) [1.97; 3.38]	<0.01
	AD grip	1.13 (0.72) [0.92; 1.11]	1.60 (0.77) [1.11; 2.09]	0.05

L grip: Lachman grip; AD grip: anterior drawer grip. Anterior tibial translation was measured in mm and presented as mean ± standard deviation (SD) for healthy subjects and anterior cruciate ligament (ACL) patients. *p* values correspond to the results from using Student's unpaired *t*-test to test for differences between the groups.

## Data Availability

The data used to support the findings of this study are available from the corresponding author upon request.
